# Patient and public involvement in the build‐up of COVID‐19 testing in Sweden

**DOI:** 10.1111/hex.13463

**Published:** 2022-03-07

**Authors:** Mio Fredriksson

**Affiliations:** ^1^ Department of Public Health and Caring Sciences Uppsala University Uppsala Sweden

**Keywords:** COVID‐19 pandemic, COVID‐19 testing, patient and public involvement, Sweden

## Abstract

**Background:**

Patient and public involvement in healthcare can be particularly challenging during crises such as the COVID‐19 pandemic.

**Objective:**

The aims of the study, which focuses on COVID‐19 testing in Sweden, were to explore (1) how, or to what extent, patients and members of the public were involved in decisions about the organization of COVID‐19 testing during the first year of pandemic and (2) whether this was seen as feasible or desirable by regional and national stakeholders.

**Methods:**

A qualitative interview study was conducted with key organizational stakeholders at three national agencies and within three Swedish regions (*n* = 16).

**Results:**

There had been no patient and public involvement activities in the area of COVID‐19 testing. The regions had, however, tried to respond to demands or critiques from patients and the public along the way and to adapt the services to respond to their preferences. The need for rapid decision‐making, the uncertainty about whom to involve, as well as a hesitation about the appropriateness of involving patients and the public contributed to the lack of involvement.

**Conclusion:**

Future studies on patient and public involvement during crises should address what structures need to be in place to carry out involvement successfully during crises and when to use activities with varying degrees of power or decision‐making authority for patients and members of the public.

**Patient or Public Contribution:**

Fifteen members of the public contributed with short reflections on the study findings.

## 1 INTRODUCTION

A crisis occurs when interests are threatened, the effect of practical actions is uncertain, and when a quick response is needed.^1^, ^2^ Leaders in a crisis such as the COVID‐19 pandemic are confronted by a number of challenges, among them the need to make decisions rapidly in spite of having limited and fragmented information, which may lead to a contraction of decision‐making authority and a reduced tolerance for ambiguity.^2^ Cattapan et al.^3^ argue that rapid and decisive action by governments can be crucial to saving lives in a time of crisis, but simultaneously constitutes a challenge to public involvement. In line with this, it was noticed in the United Kingdom a few months into the pandemic that, in the rush to introduce new COVID‐19 policies and make service reconfigurations, patient and public consultation had largely been bypassed.^4^ Mouter et al.^5^ also noticed an absence of public involvement in decision‐making about measures to curb the spread of the virus in the Netherlands. At the same time, it has been argued that patient involvement is the most powerful weapon to fight COVID‐19,^6^ that patients and members of the public should be involved in difficult discussions and decision‐making processes regarding prioritization and treatment allocations during the pandemic,^7^ and that an inclusive approach dealing with COVID‐19 should be based on the empowerment of communities.^8^

Although involving the public in policymaking during a crisis may be particularly challenging, there are a range of rationales for doing so according to Mouter et al.^5^ They present substantive, normative, and instrumental justifications for public involvement in crisis policymaking. The *substantive rationale* suggests that public involvement will improve the quality of decisions. Input through a participatory process can align decisions with the public's preferences and the public may contribute with ideas, arguments, and values outside of the decision‐makers' radar. The *normative rationale* suggests that public involvement is morally right because citizens (i.e., the public) should be able to make their voice heard in decisions that are of importance for their lives and for the community. It has been argued that health emergency policies should be participatory since they are ‘deeply affecting people in very sensitive domains’^5^ (p. 3). Lastly, the *instrumental rationale* implies that the aim is to achieve a particular predefined end, for instance increasing the acceptance of certain policies (such as when to get tested for COVID‐19), which in turn can increase compliance.

This article focuses on patient and public involvement (PPI) in COVID‐19 testing. Along with measures such as social distancing, the main pillars of the COVID‐19 public health response in Europe during 2020 were robust testing capacity with prompt isolation of cases, contact tracing, and quarantine of identified contacts.^9^ COVID‐19 testing was considered crucial to mitigate the impact of the pandemic on vulnerable populations and healthcare systems, and to ensure the continued functioning of societies and economies.^10^ The European Centre for Disease Prevention and Control (ECDC) stressed that COVID‐19 testing strategies should be flexible, and before implementing any population‐wide testing strategy, it was important to consider factors such as transmission, costs, logistics, and barriers to testing.^9^ The ECDC encouraged that testing efforts were maximized from June 2020.^11^

One of the countries that had a rather slow increase in the testing capacity per 100,000 inhabitants during 2020 was Sweden,^12^ which also differed compared to their neighbors in pandemic response. Overall, the 21 self‐governing regions responsible for funding and provision of healthcare followed the recommendations issued by the Public Health Agency of Sweden (PHA) for whom to test for the COVID‐19 infection. In early 2020, only certain individuals were tested (e.g., people coming from particular areas abroad). Between mid‐March and June, COVID‐19 testing was expanded to inpatients at hospitals, individuals belonging to a risk group, residents in care and in institutions, and health‐ and social care staff. It was only from mid‐June 2020 that the 21 regions offered tests to *all* members of the population with symptoms (population‐wide testing). Largely, the regions have been free to organize their testing systems according to local conditions, and, for example, some regions have offered home tests and drop‐in tests, while others have not. From June 2020, the regions had financial support from the government. In the last week of May 2020, 36,466 individuals were tested in Sweden compared to 232,114 individuals in the last week of 2020.

The aims of the study, which focuses on COVID‐19 testing in Sweden, were to explore (1) how, or to what extent, patients and members of the public were involved in decisions about the organization of COVID‐19 testing during the first year of pandemic and (2) whether this was seen as feasible or desirable by regional and national stakeholders.

As the regions are self‐governing, in practice, there are 21 regional health systems in Sweden and they differ in their organizational set‐up and how they carry out PPI. There are no national policy documents that prescribe how PPI should be carried out (or that it should take place) in the service‐provision part of the organization. There is however an increasing consensus about the need to make healthcare more person‐centered and the Patient Law (2014:821) establishes that the care patients receive should as far as possible be decided on and carried out in consultation with the patient.^13^ At large, public involvement is channeled through the regions' democratic system, and the Municipal Act (2017:725) establishes that the political committees in the regions should strive to consult the service users.^14^ Thus, public involvement betweenis desirable but not a requirement.

## 2 MATERIALS AND METHODS

### 2.1 Design

This is a qualitative interview study that draws on individual semi‐structured interviews with organizational stakeholders responsible for the build‐up of COVID‐19 testing in Sweden during 2020. Interviews were chosen because of the potential to gain a deeper understanding of experiences, attitudes, and processes linked to PPI in COVID‐19 testing.^15^

### 2.2 Sample and data collection

As the interviews were carried out during the third wave (February–April 2021) and health professionals and managers had a heavy workload, we concentrated on three of the regions where it was possible to recruit participants. The three regions vary in geographical and population size, which allowed for a range of potential experiences, but the regions were hit by a more extensive spread of COVID‐19 roughly at the same time and thus experienced the same high pressure to offer COVID‐19 testing. The sample consists of 16 interviews with a range of key organizational stakeholders in the build‐up of COVID‐19 testing in these regions, as well as key representatives at three national authorities (see details in Table 1). In the selected regions all invited participants agreed to participate (sometimes after one or two reminders), while two declined at the national level. The sample is narrow in the sense that it only captures PPI in relation to COVID‐19 testing. The interviews were conducted by the author and a doctoral student, who both have conducted many collections of qualitative data. The interviews were conducted by using the Zoom platform, only saving the sound file, and lasted between 45 min and 1 h.

**Table 1:** Description of the interview sample

Function	Description	Respondents
The physician responsible for disease control in the region (*sve. smittskyddsläkare*)	These physicians have an overall responsibility for disease control within the region. They plan, organize, and lead the work, for example ensuring that the public has the information it needs to protect themselves against infectious diseases. They also give advice to particularly vulnerable groups, and support healthcare staff.	#1, #2, #3
Physicians responsible for COVID‐19 testing	The testing system and the organization of testing differ between the regions. We interviewed the personnel in charge of each region's system.	#4, #5, #6, #7
Representatives at the region's central communications unit	The organization of communications differs between the regions. We interviewed key people at the information/communication units in each region.	#8, #9, #10
Representatives from the County Administrative Boards	From June, the county administrative boards were assigned by the government to assist the regions in the build‐up of testing capacity and in performing tests.	#11, #12, #13
Representative from the Public Health Agency of Sweden (PHA)	The PHA has a national responsibility for public health issues and works to ensure good public health. The agency also works to ensure that the population is protected against communicable diseases and other health threats.	#14
Representative from the Swedish Association of Local Authorities and Regions (SALAR)	SALAR is an employers' organization which represents and advocates for local government in Sweden. All of Sweden's municipalities and regions are members of SALAR.	#15
Representative from The Swedish Civil Contingencies Agency	Responsible for issues concerning civil protection, public safety, emergency management and civil defence as long as no other authority has responsibility.	#16

The interviews were made in an ongoing research project investigating how the Swedish regions handled the build‐up and expansion of COVID‐19 testing during the first year of the pandemic and how this was coordinated at the national level. At the end of the interviews, there were two broad questions about PPI in this process: more precisely about how patients or members of the public had been involved and whether the respondent saw this as possible and desirable during the first year of the COVID‐19 pandemic. The interview guide was pilot tested and revised.

The participants were contacted via email with a request for participation. Information about the study's purpose, the voluntary nature of the study, and the possibility of withdrawing at any time was attached. At the beginning of the interview, the participants were again briefly informed about the study and signed a consent form to participate. The study was approved by the Swedish Ethical Review Authority (2020‐05732).

### 2.3 Analysis

The interviews were transcribed verbatim and analyzed thematically. Under the main themes of conducted activities, feasibility, and desirability of PPI during the COVID‐19 pandemic an inductive thematic analysis was carried out.^16^ The author conducted the analysis and discussed and validated the results together with the doctoral student involved in data collection.

## 3 RESULTS

### 3.1 PPI during the first year of the pandemic

There were no systematic differences between the three regions in how PPI had been incorporated into the build‐up of the COVID‐19 testing system. The interviews show that there had been no PPI activities in the area of COVID‐19 testing during the first year of the pandemic. At least, the respondents in the regions and the County Administrative Boards did not know about any such activities.

A respondent responsible for testing in a region said that they had decided how to structure the COVID‐19 testing service in their region without consulting potential users (*we have not asked people what they want* #7). Similarly, another respondent responsible for COVID‐19 testing mentioned that their region had not had a reference group or similar when building up their testing system. This respondent expressed this in the following way:

*For example, when we expanded testing to the general public [from June 2020], we did it without asking anyone, it was more like: this is how it will be*. (#4)

The same message was conveyed by a respondent from one of the regions' communication units:

*We have not had time to have a detailed dialogue about every new service that we needed to start up during the pandemic, we have not, it has not ‘been on the map’*. (#9)

The representative from the Swedish Association of Local Authorities and Regions (SALAR) confirmed the lack of involvement and said that they had met some disappointed patient organizations and thought that the regions and SALAR had to reflect on this after the pandemic and think about how to support the involvement of different groups. A respondent from one of the county administrative boards mentioned that looking at it in retrospect, the public's perspectives on where testing stations were located, for example, should have been taken into account in the process.

Importantly, however, the respondents in the regions explained that they had considered opinions about the functioning of the COVID‐19 testing service along the way and had continuously handled problems related to the build‐up and expansion. One example was what a person should do if they lived alone, did not have a car, and could not take the bus to the testing station because they suspected they had COVID‐19. When asked whether there had been any structured follow‐ups on patient and public opinions on COVID‐19 testing during the first year of the pandemic, the respondents from the regions said there had not. A respondent responsible for COVID‐19 testing in one of the regions mentioned that they did not send out any surveys or similar, but that instead, they worked with the feedback they got from the public through emails or verbally at the testing stations. This seemed to be a common response and the regions got feedback on numerous aspects such as how quickly you got your test delivered to your home, the location of testing stations, why you had to have a Swedish personal identification number and a bank ID when booking a test, and so forth. One respondent responsible for COVID‐19 testing expressed their follow‐up like this:

*But let us put it like this, a lot is written in the newspaper, and it was a ‘damn hullabaloo’ before we got started [with general testing] and people thought it was too slow, and I can tell you that I have been in the newspaper numerous times regarding this issue, and also in radio and TV (…) But we have not asked*. (#6)

The PHA mentioned that the agency received many emails from the public with questions about the handling of COVID‐19 and that the agency had opened up a specific email service for questions about testing, where they got input from the public regarding whether the regions had sufficient testing capacity or not, for example. The agency did not, however, communicate with any specific patient groups with regard to testing.

Furthermore, several of the respondents mentioned that communication to patients and members of the public was crucial, among other things to make them understand when to get tested and what to do if they had COVID‐19 symptoms. The respondents mentioned that communication was largely handled centrally by the regions, for instance through press conferences and local radio. Furthermore, it was stated that the county administrative boards had helped to disseminate information and that the regions had worked closely with the municipalities, for instance, to reach vulnerable populations not speaking Swedish. It was also stated that certain civil society organizations were involved in translating and spreading information through channels such as Snapchat. However, one of the respondents responsible for COVID‐19 testing in a region mentioned that the region should have been better at spreading information through sports associations, churches, and so on, about the importance of being tested if symptoms had been detected. In one of the regions, it was also mentioned that Facebook had been a very important platform for communication together with 1177 (Sweden's digital platform for information and services within healthcare). Representatives from the PHA and the Swedish Civil Contingencies Agency also mentioned that a crucial part of their work during the pandemic had been to develop their communication to reach all types of groups in society. The PHA, for instance, had direct dialogues with members of the public from a disadvantaged area in the capital where it is generally difficult to reach people with messages from authorities.

### 3.2 The possibility and desirability of PPI

When discussing whether it had been possible or desirable with PPI in the build‐up of COVID‐19 testing, many of the respondents were uncertain. However, at the same time, they knew there could be benefits to PPI more broadly. One of the respondents responsible for COVID‐19 testing answered symptomatically: ‘Well, it feels weird to answer no to that question’ (#5). Another of the respondents, who was the physician responsible for disease control in a region said: ‘In that case, it is potentially thinkable that you could have some representatives as a sounding board, possibly’ (#2). One respondent stated that he/she wished they had had a panel of ‘ordinary’ members of the public already established that they could rapidly assemble to give their opinion and to test new solutions.

During this period, there were three main types of uncertainty around PPI expressed by the respondents, of which the first and most salient was the *speed* of the processes. It was repeatedly mentioned that there had been no time to consult patients or members of the public. A respondent responsible for developing testing capacity in a region said:

*I try to think back and think about when I could have got it [PPI] into the work and I can only say that it would have been window dressing, because everything went so fast, and there were so many different things that we handled at the same time. None of us worked with this issue alone and (…) we made hundreds of decisions and worked with things that did not lead to any decisions. So I think it would have been more symbolic than real*. (#7)

A respondent being the key person in his/her region's communication work expressed it like this:

*Periodically, it has been minute‐by‐minute prioritisation, we have had working days, most often, which are at least ten hours. I have had working weeks of sixty hours for months and months and I have six hundred hours of overtime. We work constantly: evenings, weekends, and then there is still no time to sit in any booked meetings with patient associations, we are in the middle of a burning crisis*. (#8)

Another type of uncertainty around involvement concerned *who should be involved*, which some of the respondents said that they did not see clearly, since COVID‐19 is a disease affecting everybody. One of the respondents responsible for COVID‐19 testing said that there was no user organization to talk to because it concerned everyone, and that they did not see whom they could have involved: ‘I don't know: who would be the discussion partner except for the politicians?’ (#6). The same respondent mentioned that they had been in close contact with the politicians in charge, which he/she saw as the extended arm of the members of the public.

The third type of hesitation expressed by some of the respondents regarded *the participants' contribution*. For example, a respondent responsible for COVID‐19 testing in one of the regions pointed out that he/she did not think involvement would have changed the situation at all, at least not in the beginning of the pandemic when the big problem was a lack of testing supplies and laboratory capacity for analyzing tests. Another respondent said that because the PHA provided guidelines for whom to test, it was unclear what patients or members of the public could have influenced. When discussing whether the public could have been involved in decisions about where to set up testing stations in one of the regions, the physician responsible for disease control expressed the problem of securing a broader perspective:

*No I don't really see it, then everyone wants the test‐station close to where they are (…) it is always about prioritizing your own group, which makes it difficult with too extensive involvement*. (#1)

Several other respondents expressed themselves similarly, and one of the respondents responsible for communication in a region pointed to an established order of expertise that was followed when it came to COVID‐19 testing, which did not include patients or members of the public:

*It is very much an issue for the physician responsible for disease control in the region, an expert role, how to do in a crisis situation. It is not the case that you go and ask a patient association how to do the testing*. (#8)

However, some respondents expressed a more supportive attitude. For example, one respondent mentioned that to be able to communicate effectively, it is important to understand people's intentions. Another one meant that some of the problems with logistics could probably have been solved earlier if more diverse perspectives had been included in the process, for example, problems related to the need for a bank ID to be able to book a test and testing options for asylum‐seekers.

### 3.3 Short reflections by members of the public

To improve the interpretation and understanding of the results, a number of members of the public were invited to contribute with an individual short reflection on the study findings, that is, that no PPI was carried out, and the reasons given by the national and regional stakeholders. Twenty members of the public were invited through contacts in the three regions and an invitation was also conveyed in a group of patients engaged in another type of PPI activity. In total, 15 individuals, aged between 18 and 83 years and living in rural as well as urban parts of the three regions, contributed in a telephone conversation or by written reflections.

Overall, the members of the public expressed an understanding that the lack of time did not make PPI possible. Someone questioned if decisions would really have become better with more involvement because it concerned such complex and technical issues. A few wondered if more involvement really could have solved problems such as the lack of testing‐ and analysis capacity, which were the main bottlenecks. Several members of the public meant that this is why we have experts, to make this type of decision. In general, the members of the public expressed a high level of confidence in experts. However, it was also mentioned that more involvement could have contributed with a deeper understanding of different perspectives, for instance, those of older people without a bank ID (which was needed to book a test), people with disabilities, and with difficulties understanding Swedish. For example, a woman in her 40s said that there had been many problems linked to booking a COVID‐19 test for children over 13 years of age (which required a bank ID that most children did not have) and that this would probably have been solved with public involvement. Members of the public thought that aspects of testing that could have been improved with more involvement were access to testing, the information on how to get tested (particularly concerning school children), the number and location of testing stations, and whether the region offered home tests or not.

Some of the members of the public thought that it was less understandable that those responsible in the three regions did not know whom to involve and did not already have established channels for consulting patients and members of the public. This signaled that they were not used to involving patients or members of the public. A man in his 30s expressed that it was worrying from a democratic point of view and several of the members of the public mentioned that there must have been opportunities to quickly consult with, at least, some patient organization. A man in his 70s thought the regions should use some digital solution to be able to quickly survey the public's opinions and a woman around the age of 20 hoped that the regions would improve their PPI after the pandemic.

## 4 DISCUSSION

Most studies on PPI at the levels of organizational design and governance, or policymaking, investigate particular PPI activities or PPI fora, for example how they work, who is represented, and their impact. In this article, however, PPI in the decisions about the organization of COVID‐19 testing in Sweden was investigated—a service that did not exist before the pandemic but which was emphasized as crucial for a country's COVID‐19 response by the WHO and the ECDC.^10^, ^17^ This means how PPI, in general, has been carried out in the Swedish regions during the pandemic was not explored.

Overall, the interviews show that there had been no PPI in the build‐up of the COVID‐19 testing system in the three regions being the focus of the study. Similarly, in a scoping review, Cadel et al.^18^ found few examples of involvement in organizational level decision making (design of care) during the first 6 months of the COVID‐19 pandemic. The regions had, however, tried to respond to demands or critique from patients and the public along the way and to adapt the services to meet their preferences. Thus, in terms of the continuum of engagement, or involvement, described by Carman et al.,^19^ there had been no planned *consultation* and thus no structured opportunity for patients or members of the public to express views or contribute with opinions on the build‐up and expansion of COVID‐19 testing, which is considered to be the lowest end of the continuum. *Information* is often added to this continuum, and our results show that the three regions and the national authorities had worked rather extensively to communicate their message to patients and members of the public, for example about when and how to be tested for COVID‐19. There had been specific efforts to communicate with ‘hard‐to‐reach’‐groups not speaking Swedish and that are generally difficult to reach with messages from authorities because of relatively low levels of literacy and being outside the labor market.

The main reason why the respondents thought it was not feasible to involve patients and the public in the build‐up and organization of COVID‐19 testing was the need to make decisions quickly (time being a PPI facilitation barrier).^20^ This corresponds with literature predicting a contraction of decision‐making authority to speed up processes during a crisis, which constitutes a challenge to public involvement.^2^ The lack of time during a crisis makes it difficult to give participants the proper training and support, which has been pointed out as necessary for successful involvement.^21^, ^22^ All members of the public reflecting on the study findings however accepted that the lack of time was an obstacle.

Another reason why involvement was difficult was that the professionals being responsible for setting up the systems did not know whom to involve, since there was no particular patient group that was affected, and they had no established forum of patients or members of the public to consult. They did not see testing as an issue to discuss with patient organizations as it was not patient‐specific. Uncertainties about practicalities and the imprecise role of patients and the public is another facilitation barrier described in the literature,^20^ and it has been suggested that it may be advisable to have a citizen jury institutionalized as part of a public health response plan.^23^ Similarly, one respondent wished they had already established a panel of ‘ordinary’ members of the public that they could rapidly assemble, and when reflecting on the study findings several members of the public expressed some concern that such channels did not exist.

In addition, there was some hesitation about the desirability of PPI in these types of decisions during a crisis. Some organizational stakeholders expressed a positive attitude to PPI more broadly whereas others expressed more of a restrictive approach, also sharing a concern for opening up for too much self‐interest in important decisions.^24^ There was also some hesitation about the participants' potential contribution, although involvement could be seen as important to investigate factors such as logistics and barriers to testing, which the ECDC stated should be considered before implementing any population‐wide COVID‐19 testing strategy.^9^ A perceived superiority of professional knowledge^25^ could also be detected in some of the respondents. The same beliefs and reliance on expert knowledge were also expressed by some of the members of the public when reflecting on the study findings.

Hickey and Chambers^26^ have suggested that involvement challenges are related to the cultures of organizations and their processes and to professional identity (professionals may feel that their role will be undermined).^27^ Nevertheless, it has been argued that without PPI ‘researchers and clinicians may ultimately miss the needs deemed as high priority by the end users’,^20^ and in the case of COVID‐19 testing, this could have applied to issues like the localization of testing stations, whether the region should offer home‐tests or not, or how the system for booking a COVID‐19 test was designed. All these are examples of issues that members of the public reflecting on the study findings thought their additional perspective could have made a contribution to. This reasoning links back to the justifications for public involvement in crisis policymaking presented by Mouter et al.^5^ However, in the interviews, it was difficult to identify the reasoning behind the substantive, normative, and instrumental rationales.

According to Mouter et al.^5^ opportunities for input from the public are easily lost during a crisis if there is not a high degree of determination in the organization. Our results suggest that such a determination did not exist with regard to COVID‐19 testing, indicating a lack of PPI culture,^27^ which can possibly be linked to a lack of policy and guidance on how to carry out collective forms of PPI at the service‐provision level within the Swedish healthcare system. Today, there is a large variation in how hospitals and other healthcare facilities or clinics carry out PPI. Some hospitals or hospital clinics have a patient and family council while a ‘suggestion box’ is probably most common, or invitations to patients to contact the statutory patient committee with questions, viewpoints, or complaints. According to de Graaff et al.,^28^ initiating and sustaining involvement initiatives require considerable effort. This suggests that if activities or involvement fora have not been established and developed under more ordinary conditions, they may be impracticable during a crisis. Similarly, Leese et al.^29^ point to the importance of having established trusting relationships before a crisis.

de Graaff et al.^28^ have also argued that ‘it is time to move beyond discussing patient and public involvement and engagement (PPIE) as something that we can never have enough of and to start examining more thoroughly the work necessary to make PPIE work in healthcare decision‐making’. By that, they mean that activities should be tailor‐made (in terms of whom to involve, how to involve them and how to value their contributions) and that an involvement method's effect largely depends on the situation. Still, little is known about what works during crises. Poorly planned or performed PPI, or ‘tokenism’, may reduce trust and undermine the benefits of true involvement.^20^ At the same time, it is likely that a lack of PPI impairs trust and legitimacy. Future studies of PPI during crises should address what structures need to be in place to carry out PPI successfully during crises. How can patients and members of the public be involved in a meaningful way when there is time pressure, lack of information, high demand for services, and extensive media coverage? These study could address if less comprehensive involvement activities can fill important functions during crises or if it requires long‐standing public consultation processes that build trust and capacity and that keep lines of communication open.^3^, ^30^ It could also address equity challenges in the use of technology to carry out PPI during crises.^18^

Lastly, there are some limitations to this study. Because the interviews were carried out during a time of heavy workload of potential respondents (the third wave), the number of respondents was relatively small and had to be drawn from regions where it was possible to recruit participants. This may have led us to miss important aspects of PPI in other regions. There was, however, a high level of saturation in the respondents' descriptions of PPI in COVID‐19 testing during the first year of the pandemic although the regions varied in size and geographical conditions. To what extent the results are generalizable to other settings is uncertain, but since the respondents across the three different‐sized regions conveyed the same picture of PPI in COVID‐19 testing, it is likely that this applies to COVID‐19 testing in Sweden at large. Furthermore, no patients or members of the public were interviewed about their role in the organization of the COVID‐19 testing system.

## 5 CONCLUSION

There was no PPI in the build‐up of the COVID‐19 testing service in Sweden during the first year of the pandemic although there are substantive, normative, and instrumental justifications for involvement during crises. However, service adaptions were made as a response to incoming communication from patients and members of the public. The need for rapid decision‐making, the uncertainty about whom to involve, and hesitation about the appropriateness of PPI and its contribution were reasons for the lack of PPI. Thus, in Sweden PPI is ‘still largely seen as “nice to have”, but not essential’.^7^ Future studies on PPI during crises should address what structures need to be in place to carry out PPI successfully during crises and when to use activities with varying degrees of power or decision‐making authority for patients and members of the public.

## Data Availability

No data were available.
